# Bacterial Exopolysaccharides: Functionality and Prospects

**DOI:** 10.3390/ijms131114002

**Published:** 2012-10-30

**Authors:** Uchechukwu U. Nwodo, Ezekiel Green, Anthony I. Okoh

**Affiliations:** Applied and Environmental Microbiology Research Group (AEMREG), Department of Biochemistry and Microbiology, University of Fort Hare, Private Bag X1314, Alice 5700, South Africa; E-Mails: egreen@ufh.ac.za (E.G.); aokoh@ufh.ac.za (A.I.O.)

**Keywords:** exopolysaccharides, biopolymers, glycocongugates, extracellular, antigenicity

## Abstract

Diverse structural, functional and valuable polysaccharides are synthesized by bacteria of all taxa and secreted into the external environment. These polysaccharides are referred to as exopolysaccharides and they may either be homopolymeric or heteropolymeric in composition and of diverse high molecular weights (10 to 1000 kDa). The material properties of exopolysaccharides have revolutionized the industrial and medical sectors due to their retinue of functional applications and prospects. These applications have been extensive in areas such as pharmacological, nutraceutical, functional food, cosmeceutical, herbicides and insecticides among others, while prospects includes uses as anticoagulant, antithrombotic, immunomodulation, anticancer and as bioflocculants. Due to the extensive applications of bacterial exopolysaccharides, this overview provides basic information on their physiologic and morphologic functions as well as their applications and prospects in the medical and industrial sectors.

## 1. Introduction

Bacteria produce diverse biopolymers with varied chemical properties via utilization of simple to complex substrates. Some of these biopolymers serve the same function whereas others are specific for certain taxa and serve distinct biological functions [1,2]. With respect to cellular location, biopolymers could either be intracellular or extracellular. The intracellular biopolymers are few and have very limited use; however, the range of the extracellular biopolymers are vast and may be grouped into four major classes; polysaccharides, inorganic polyanhydrides (such as polyphosphates), polyesters, and polyamides [3,4], and have been collectively termed extracellular polymeric substances [3], slime and microcapsular polysaccharides [5,6] among others. Their functions includes adherence of cells to surfaces, migration of prokaryotes in groundwater, protection from engulfment by predatory protozoa and white blood cells (phagocytes), protection from perennial effects of drying or desiccation in certain soil bacteria or from attack by antimicrobial agents of plant or animal origin and the communal life of biofilm [7]. Nichols *et al.*[8] and Junge *et al*. [9] suggests functions which includes cryoprotection for growth at low temperatures and high salinity with reference to sea ice microbial community and bacteria of other marine environments (Antarctic and soda lakes among others).

Polysaccharide components of the extracellular biopolymers are the most abundant [10] and their location relative to the cell, again, forms the basis for their classification. At the cell wall, they serve structural and protective purposes and are found as constituents in teichoic acids. Outside the cell, they may take the form of a covalently bound cohesive layer; a morphologic entity termed capsule [6] or completely excreted into the environment as slime [3]. Often these capsules serve as adherents of cells to surfaces and may be overproduced when there is abundance of sugars to become reserves of carbohydrate for subsequent metabolism [11,12]; dextran is a good example in this group. Nonetheless, the distinction between loosely attached and unattached extracellular polymeric substance lies in the structural and functional relationship with the cell.

Technological advancement has led to discovery of the usefulness of bacterial biopolymers to man, consequently a myriad of industrial and medical applications ensured. The inherent biocompatibility and apparent non-toxic nature of some of these bacterial exopolysaccharides has prompted their uses in numerous medical applications; as scaffolds or matrices in tissue engineering, drug delivery and wound dressing, thus making them more attractive as compared to polysaccharides obtained from plants and microalgae [4,13,14]. Some biopolymers are gradually degraded *in vivo*, making them well suited for use in tissue replacement and controlled drug release [4]. This overview provides recent advancements in the knowledge of functional properties of bacterial exopolysaccharides, applications in medical and industrial sector and their future prospects.

## 2. Morphologic and Functional Properties of Bacterial Exopolysaccharide

The history of bacterial exopolysaccharides began during the mid-19th century with the discovery of an exopolysaccharide in wine, which would later be known as dextran and the prokaryote responsible for the production was identified as *Leuconostoc mesenteriodes*[4,15]. Over the course of time, other exoplysaccharides discovered includes cellulose, alginate and xanthan. Advances in science led to the use of bacteriolytic enzymes and radioisotope labeling of precursors for biosynthetic studies thus, some details about the metabolic pathways for biopolymer formation were elucidated [16]. An instance is in the elucidation of capsular polysaccharide from Klebsiella K15 using 1D and 2D ^1^H and ^13^C NMR spectroscopy of the alditol (an oligosaccharide) obtained by depolymerisation of the polysaccharide with a viral-borne endoglycanase [17]. Consequently, the structural and functional roles of the prokaryotic exopolysaccharides gradually came to light.

### 2.1. Bacterial Capsule

Layers of surface-associated covalently bound polysaccharides, anatomically positioned as the outermost covering of bacteria cells are referred to as capsule. They impart mucoid appearance to bacterial colonies grown on laboratory agar media. The nature and composition of capsular polysaccharides is very much strain dependent [18], and the structural diversity shown, is reflected in the more than eighty different types known in *Escherichia coli* alone. However, some types are shared by bacteria from different taxa, and a good example is the *E. coli* K1 and *Neisseria meningitidis* B capsules which contains sialic acid [19]. Some bacteria are taxonomically grouped based on their capsular polysaccharides and this is exemplified in *E. coli* were *O*-antigen; cell wall derived, *H*-antigen; flagella derived, and K-antigen; capsule derived forms antigenic classification of the organism. The *K*-antigen is further divided into L, B and A groups [20]. Capsules provide multiple functions to the bacterial cells, which include, as adhesion receptors during colonization of tissues, acting as a deterrent to desiccation because they attract water. Moreover, the multiple variation in structure confers resistance to various phages and vertebrate complement.

### 2.2. Exopolysaccharides

Bacterial polysaccharides synthesized and secreted into the external environment or are synthesized extracellularly by cell wall-anchored enzymes may be referred to as exopolysaccharides. The overwhelming diversity of bacterial polysaccharides allows for categorization based on chemical structure, functionality, molecular weight and linkage bonds. Following the chemical composition, exopolysaccharides may be looked at based on monomeric composition and as such; homopolysaccharides and heteropolysaccharides are the two groups recognized [6]. Homopolysaccharides contain only one type of monosaccharide while heteropolysaccharides is composed of repeating units, varying in size from disaccharides to heptasaccharides.

Exopolysaccharide categorization are complex and in some instances characterization factors are reapplied so as to further make distinctions between groups and this is seen in homopolysaccharides been further clustered into four groups thus; α-d-glucans, β-d-glucans, fructans and polygalactan [21]; this grouping is based on linkage bonds and nature of monomeric units. On the other hand, the composition of heteropolysaccharides includes the repeating units of d-glucose, d-galactose, l-rhamnose and, in a some instance, *N*-acetylglucosamine (GlcNAc), *N*-acetylgalactosamine (GalNAc) or glucuronic acid (GlcA). Non-carbohydrate substituent such as phosphate, acetyl and glycerol are sometimes present [6]. Bonds between monomeric units at the backbone of the polymers are 1,4-β- or 1,3-β-linkages and 1,2-α- or 1,6-α-linkages. The former is characterized by strong rigidity while the latter; more flexible ones. The differences between homopolysaccharide and heteropolysaccharide are not only reflected in the chemical nature and linkage bonds but in synthetic enzymes and site of synthesis. The precursor repeating units of heteropolysaccharide are formed intracellularly and isoprenoid glycosyl carrier lipids are involved in translocation of the precursors across the membrane for subsequent polymerisation extracellularly [22], whereas homopolysaccharides syntheses require specific substrate such as sucrose. Furthermore, the quantity of exopolysaccharides produced varies with bacteria species. However, the physicochemical factors playing crucial role in the yield of these compounds includes pH, temperature, incubation time (laboratory conditions), and medium composition (carbon, nitrogen and cation sources) [23]. However, it is not clear if the chemical nature or monomeric compositions of heteropolysaccharides are influenced by carbon and nitrogen sources, unlike homopolysaccharides.

Exopolysaccharide have similarly been categorized on functionality, and as such seven categories were proposed by Flemming *et al*. [24]; constructive or structural, sorptive, surface-active, active, informative, redox-active and nutritive exopolysaccharides respectively. Nonetheless, Flemming *et al*. [24] put forward a concept which advances that the classification is not exclusively inclusive as groupings like exopolysaccharides involved in biocide resistance are not captured. In essence, this is a field where a lot has been done, yet more work is still needed. In the light of the above grouping, biomolecules classified as structural exopolysaccharides includes neutral polysaccharides as they serve architectural purposes in the matrix facilitating water retention and cell protection. Surface active exopolysaccharides includes molecules with amphiphilic behavior; they have varied chemical structures and surface properties and may be involved in biofilm formation and/or sometimes possess antibacterial or antifungal activities. Sorptive exopolysaccharides are composed of charged polymers, whose function is sorption to other charged molecules involved in cell-surface interactions [24,25].

### 2.3. Important Polysaccharides from Marine Bacteria

Marine bacteria offer a great diversity of polysaccharides which could play an important role in biotechnology and industry as well as in future development of cell therapy and regenerative medicine among others applications. The wealth and diversity of the marine biosphere which includes the deep sea hydrothermal vents, Arctic and Antarctic sea ice has not been fully explored hence, great prospects abound for discovery of novel polysaccharides.

*Spirulina* has been used as pharmaceutical additives and for nutritive purposes with no risk to health. Furthermore, studies suggest that compounds basically composed of polysaccharides found in *Spirulina* have antiflammatory properties amidst other therapeutic functions [26]. Additionally, Spirulan; a sulfated polysaccharide produced by *Arthrospira platensis* (formely *Spirulina platensis*), has been documented as an inhibitor of pulmonary metastasis in humans and a preventer of adhesion and proliferation of tumor cells. To this end, electrospining biomass of *Spirulina* to porous scaffolds and nanofibers are concepts developed for the treatment of spinal cord injury [27,28]. Similarly, the marine bacteria; *Vibrio diabolicus* produces polysaccharides that are hyaluronic acid like and have been commercialized with “Hyalurift” trade name. The polysaccharide has been shown to have restoration activity to bone integrity [28].

## 3. Exopolysaccharides in Bacterial Biofilm

In nature, bacteria exists in colonies accumulating at interfaces to form poly-bacterial aggregates such as mats, flocs, sludge or biofilms and not planktonic dispersed single cells as will be seen in laboratory pure cultures [12]. Bacteria are not alone in this endeavor as other microbes are inclusive. However, our emphasis is with respect to bacteria and how their exopolysaccharides play crucial roles. Furthermore, for less ambiguity we will stick to the use of term biofilms to mean microbial aggregates that accumulate at a solid–liquid interface and are encased in a matrix of highly hydrated extracellular biopolymers. Although this description does not take into account groups of free floating microbial aggregates (flocs).

Biofilms have been metaphorically dubbed “city of microbes” [29], and the extracellular biopolymers, in which exopolysaccharide predominates, as the “house of the biofilm cells” [24], Furthermore, *Pseudomonas aeruginosa* has been referred to as the *Escherichia coli* of biofilm research, because it is the most investigated bacteria with respect to biofilms research [12]. Biofilms have been extensively studied [29–31], and a summary of the roles played by exopolysaccharides in bacterial biofilms is articulated in Table 1 and some human diseases involving biofilms are summarized in Table 2.

## 4. Bacterial Exopolysaccharides Antigen

Bacterial exopolysaccharides are contextually limited to all forms of polysaccharides synthesized and secreted into cellular external environment which may remain loosely attached to the surface (capsule) or completely detached. Polysaccharide capsular constituents (polysaccharides and/or glycol-conjugates of protein and lipids) represents major surface antigens for slimy bacteria and their role in pathogenicity have been extensively investigated [32]. However, due to the great diversity shown by the exopolysaccharides with respect to monomeric units, linkages, and unique structures, varied immunogenic responses are elicited and these antigenic properties are inclusive in serologic grouping of bacteria [32]. This is seen in *Enterobacteriaceae* where over 80 different serotypes of *E. coli* have been identified [24] based on capsular polysaccharide antigen (*K* antigens).

Capsular polysaccharide antigenicity cuts across Gram status divide; this is reflected in *N. meningitidis*, *E. coli* and *Salmonella typhi* (Gram-negatives) and *Staphylococcus* spp. and *Streptococcus* spp. (Gram-positive). Capsular polysaccharide based bacterial serotyping is predicated on reactivity of specific antibodies, often generated in animals, using reference strains of particular species, with the culpable bacteria. The polysaccharides structural diversity leads to various kinds of antibody reactivities as reflected in the large numbers of serotypes found within bacteria of the same species. Table 3 shows some clinically important bacteria, associated diseases, nomenclature of capsules and the number of identified serotypes based on capsular polysaccharides. Epidemiologically, bacterial serotyping has been of great importance because it is a simple and rapid procedure and comes handy in disease outbreaks. In epidemics, it is important to monitor the spread of causative agents as certain diseases caused by some bacterial species may be limited to a few serotypes. Structural elucidation of bacterial surface polysaccharides and advances in immunology has led to the development of capsular polysaccharide based vaccines [33], which has been largely successful in combating infectious disease.

However, the limitations in this endeavor include: the complexity of surface polysaccharides within bacteria of the same species results in a large degree of antigenic variation; secondly, the homology between some bacterial surface polysaccharides and that on human cell surface translates to poor immunogenicity with further consequences as autoimmunity, and a good example would be seen with *N. meningitidis* serogroup B and human foetal neuronal cells [34]; lastly, the lack of T-lymphocyte memory nature of the polysaccharide antigen makes them poor immunogens with consequences such as restriction and delayed ontogeny of isotypes [35].

## 5. Applications of Bacterial Exopolysaccharides

The discoveries of numerous types of exopolysaccharides have been documented. However, only a handful have been shown to have industrial and medical relevance with significant commercial value, particularly with regard to their use as biomaterials [36] or as rheology modifiers of aqueous systems. The limitation of the applications of some of these bacterial polysaccharides has been largely due to cost of production relative to their commercial value; however the approach generally employed to address this problem includes; using cheaper substrates, improving product yield by optimizing fermentation conditions, or developing higher yielding strains via mutagenesis, and/or genetic and metabolic manipulations, and optimizing downstream processing [4]. Conversely, the possession of unique properties by the exopolysaccharides, which may not be found in other traditional (plant and algae) polysaccharides would invariably translate to high-value applications thus, product quality wholly surpasses production cost [36]. Advances in the application of bacterial exopolysaccharides in medicine and biotechnology have seen uses to include bacterial alginate in cell microencapsulation, such as microsphere vectors for drug delivery, making dental impressions, as an active ingredient in absorbent dressings, and anti-reflux therapies [37]. Likewise, dextran, produced by *Leuconostoc mesenteriodes*, have been used to prepare one of the most effective plasma substitutes for application in shock and the loss of blood [38]. Glycosaminoglycan heparin, the drug of choice in the prevention and treatment of thromboembolic disorders, have been associated with inefficacy in antithrombin deficient patients with side effects as bleeding and thrombocytopenia. Thus, sulphated forms of alginate have been thought to serve as an alternative with enhanced activity. Nonetheless, other therapeutic activities attributed to sulphated forms of alginate includes; anticoagulant, antithrombotic, anti-atherosclerotic, anti-angiogenesis, anti-metastatic and anti-inflammatory [39]. Xanthan gum produced by *Xanthomonas campestris* enjoys broad industrial application. Industrial applications are broad and include areas such as in foods, toiletries, oil recovery, cosmetics and as water-based paints among other. Superior rheological properties shown by xanthan gum allow it to be used as rheological control agent in aqueous systems and as stabilizer for emulsions and suspensions [40]. In the agriculture sector, the flow ability in fungicides, herbicides, and insecticides has been improved by the addition of xanthan to uniformly suspend solid component in formulations [41]. Pesticide cling and permanence has also been noted to improve due to rheological properties of xanthan. Furthermore, ability to disperse and hydrate rapidly as well as non-pollution and good color yield status attributed to xanthan have ensured its use in jet injection printing. In a bid to go green, formulation of new generations of thermo-set coatings has included xanthan gum as it is very environmentally friendly. Similarly, the petroleum industry uses xanthan gum in oil drilling, fracturing and pipeline cleaning [40], and due to its excellent compatibility with salt and resistance to thermal degradation, it is also useful as an additive in drilling fluids. The functions and properties of a few other bacterial exopolysaccharides have been summarized (Table 4).

Microbial glycosaminoglycans (GAG) are capsular polysaccharides produced by *Escherichia coli* K5, *E. coli* K4, and *Pasteurella multocida*. GAGs are structurally linear polysaccharides, composed of repeating disaccharide units derived from amino sugars (glucosamine or galactosamine), hyaluronan, chondroitin and heparan sulphate with uronic acid as the other component of the disaccharide repeat [42]. The biological functions of GAGs includes molecular camouflage for pathogenic bacteria [43,44], however, these polysaccharides have similar backbone structure as the commercial heparin and have been synthesized by *Escherichia coli* K5 in the non sulphated forms and in the sulphated forms in *E. coli* K4. *P. multocida*, similarly, produces heparan sulphate with molecular weights between 200 and 300 kDa [44], which is higher than those of *E. coli* K5 and *E. coli* K4. GAGs are shed into the environment (fermentation medium) hence; their recovery and modification into biologically active GAGs, such as heparin or heparosan sulphate will serve as an important substitute for animal derived GAGs [45]. Important medicinal products may be obtained from GAG producing bacteria as is the situation with Group C Streptococcus (GCS). GCS serves as an important commercial source of hyaluronan polysaccharide for surgical, ophthalmic and viscoelastic applications.

## 6. Future Prospects for Bacterial Exopolysaccharides

The beneficial effects of bacteria to human health, with respect to the development of functional food, have largely been attributed to its exopolysaccharides. Some of these bacteria are referred to as probiotics; a concept Salminen *et al.*[46] describes as live microbial food ingredients which are of benefit to human health. The health promoting effects of probiotics has been attributed partly to exopolysaccharides. Antitumor, antiulcer, immunomodulatory, antiviral and cholesterol lowering activities [6,30] are some of the health benefits adduced to these exopolysaccharides. *Lactobacillus lactis* subsp. cremoris, enjoys wide application in dairy industries for yogurt production due to the special rheological properties that it impacts on products; however, this same organism is thought to possess some health-promoting properties.

Other implicated Lactobacillus strains includes *Lactobacillus delbrueckii* subsp. bulgaricus, *Lactobacillus helveticus* and *Lactobacillus rhamnosus*[47,48]. Furthermore, studies by Martin *et al*. [49] show that some bacterial exopolysaccharides alone or as conjugates could act as a very potent somnogen, thus sleep induction with a natural product with no side effects will eliminate dependence on xenobiotics for sleep induction.

Although bacterial exopolysaccharide applications spans through areas such as the industry (textile, dairy, cosmetics, *etc*.), health (medicine and pharmaceuticals) and environment (remediation, flocculation *etc*.); its application in the flocculation process will be a significant milestone to health promotion and eco-friendly usage especially in municipal and wastewater treatment processes. The flocculations of suspended particles in water treatment plants have applied the use of inorganic salts of aluminium such as aluminium sulphate and poly-aluminium chloride and organic synthetic polymers of polyacrylamide derivatives and polyethylene imine [50]. These flocculants have been shown to possess adverse health effects such as neurotoxicity, carcinogenicity and Alzheimer’s disease [51], necessitating the need for safe alternatives. This quest has been the driving force behind the investigation of the flocculating properties of some bacterial products (exopolysaccharides and other biopolymers). From our group of studies such as Cosa *et al*. [52], Piyo *et al*. [53] and Mabinya *et al*. [54] among others have reported high flocculation efficiency mediated by the biopolymers produced by *Virgibacillus* sp. Rob, *Bacillus* sp. Gilbert and *Artrobacter* sp. Raats which were fresh water and marine water isolates. The flocculation mediating biopolymers of *Virgibacillus* sp. Rob and *Bacillus* sp. Gilbert was composed of saccharide moiety as it is basically carbohydrates while *Arthrobacter* sp. Raats demonstrated about 25% polysaccharide content. These findings imply that bacterial exopolysaccharides effectively mediate flocculation and thus may be applied in large scale industrial processes, with particular reference to water and wastewater treatment.

## 7. Concluding Remarks

Bacteria exopolysaccharides shows great diversity and functions, and its production is not limited by taxa. Some of this diversity is seen in the monomeric compositions, linkage bonds and associated conjugates while the functions could be summarized as intrinsic and applied. The intrinsic functions, including morphological, structural and protective functions while applied is seen in human usage; medical, cosmetics, pharmaceutical, dairy products and other forms of industrial and environmental applications. Although a myriad of application are available for the constellation of exopolysaccharides produced by bacteria, it is vital with respect to human usage that the exopolysaccharides meet GRAS (generally regarded as safe) status or at least have a cost effective means of neutralizing toxic constituents in cases of environmental applications such as in water (waste and municipal) treatment. Production cost, largely, has been a limiting factor in the non-industrial applicability of several prospective exopolysaccharides and this is exemplified in the bio-flocculation process. However, the search for bacteria with high exopolysaccharides and exopolysaccharides conjugate (bioflocculants) yields is an ongoing process [54], while the manipulation of fermentation conditions, genetic and metabolic engineering as well as the exploration of cheap fermentation substrates for their production are suggested tools for improving the chances of commercial scale production and field application of these compounds, and these are the subject of ongoing investigations in our group.

## Figures and Tables

**Table 1:** Some of the roles ascribed to exopolysaccharides in biofilms.

Process	Functional Relevance of Exopolysaccharides to Biofilms
Adhesion	Exopolysaccharides makes provision for the initial steps in the colonization of surfaces (abiotic and biotic) and long-term attachment of biofilms.
Bacterial cell aggregation	The bridging between cells is enabled by exopolysaccharides, thus temporarily immobilizing bacterial population thus, the subsequent development of high cell densities and cell–cell recognition.
Water retention	Hydrophilic exopolysaccharides have high water retention ability thus maintaining a hydrated microenvironment around biofilm and this leading to the survival of desiccation in water-deficient environments.
Cohesion of biofilms	Neutral and charged exopolysaccharides forms a hydrated polymer network (the biofilm matrix), mediating the mechanical stability of biofilms (often in conjunction with multivalent cations), determining biofilm architecture, as well as allowing cell-cell communication.
Nutrient source	Exopolysaccharides serves as source of carbon, nitrogen and phosphorus containing compounds for utilization by the biofilm community.
Protective barrier	Exopolysaccharides confers resistance to non specific and specific host defences during infection, confers tolerance to various antimicrobial agents, protects cyanobacterial nitrogenase from the harmful effects of oxygen and offers protection against some phagocytic protozoa.
Sorption of organic Compounds and inorganic ions	Charged and hydrophobic exopolysaccharides mediates the accumulation of nutrients from the environment, sorption of xenobiotics and recalcitrant materials. They promote polysaccharide gel formation resulting in ion exchange, mineral formation and the accumulation of toxic metal ions (thus collectively contributing to environmental detoxification).
Binding of enzymes	Non glycolytic extracellular enzyme interaction with exopolysaccharides leads to retention stabilization and accumulation.
Export of cell components	Lipopolysaccharides (isoprenoid glycosyl carrier lipids), which lipo-glyco conjugate, mediates the releases cellular material as a result of metabolic turnover.
Sink for excess energy	Exopolysaccharides stores excess carbon under unbalanced carbon to nitrogen ratios.

**Table 2:** Some human disease associated with bacteria biofilms.

Human Disease	Biofilm Bacteria
Cystic fibrosis pneumonia	*P. aeruginosa* and *Burkholderia cepacia*
Otitis media	*Haemophilus influenzae* (Non-typable strains)
Periodontitis	Gram negative anaerobic oral bacteria
Dental caries	*Streptococcus* spp. and other acidogenic Gram positive cocci
Musculoskeletal infections	*Staphylococci* and other Gram-positive cocci
Necrotizing fasciitis	Group A *streptococci*
Bacterial prostatitis	*E. coli* and other Gram-negative bacteria
Urinary catheter cystitis	*E. coli* and other Gram-negative rods
Biliary tract infection	*E. coli* and other enteric bacteria
Meloidosis	*Pseudomonas pseudomallei*

**Table 3:** Some clinically important bacteria with its pathogenic serogrouping sequel to their capsule.

Bacteria Species	Pathogenic Serotypes	Capsular Antigen Nomenclature	Associated Clinical Disease
*E. coli*	>80	*K*-antigen	Diarrhoea, Neonatal meningitis and Urinary tract infection.
*H. influenzae*	>6	(a–f)	Meningitis, Epiglottitis, Septicaemia and Pneumonia.
*N. meningitidis*	>10	*K*-antigen	Meningitis, Meningococcemia.
*K. pneumonia*	>80	*K*-antigen	Pneumonia, Bacteremia, Thrombophlebitis, Urinary tract infection (UTI), Cholecystitis, Diarrhea, Upper respiratory tract infection, Wound infection, Osteomyelitis, Meningitis and Pyogenic liver abscess.
*Streptococcus pneumoniae*	>96	CPS	Otitis media, Bronchopneumonia and Meningitis.
*Staphylococcus aureus*	>11	CP	Furuncles and carbuncles, Staphylococcal scalded skin syndrome, Septic arthritis, Staphylococcal endocarditis and Atopic dermatitis.

**Table 4:** Properties and functional attributes of some bacterial exopolysaccharides.

Bacteria Exopolysaccharide	Polysaccharide Component	Molecular Weight (Da)	Properties	Applications	Bacteria strains
Dextran	Glucose	10^6^–10^9^	Non-ionic, good stability Newtonian, fluid behavior	Foods, Pharmaceutical industry (Blood volume expander) and Chromatographic media	*L. mesenteriodes*
Alginate	Guluronic acid and mannuronic acid	(0.3–1.3) × 10^6^	Gelling capacity, film forming	Food hydrocolloid and medicine (surgical dressings, wound management and controlled drug release)	*P. aeruginosa* and *A. vinelandii*
Xanthan	Glucose, mannose and glucuronic acid	(2.0–50) × 10^6^	High viscosity, Stable over a wide temperature, pH and salt concentrations ranges	Foods, petroleum industry, pharmaceuticals, cosmetics and personal care products	*Xanthomonas* spp.
Curdlan	Glucose	5 × 10^4^–2 × 10^6^	Gel-forming ability, water insolubility, edible and non-toxic has biological activity	Foods, pharmaceutical industry, heavy metal removal and concrete additive	*Rhizobium meliloti* and *Agrobacterium radiobacter*
Cellulose	Glucose	~10^6^	Not soluble in most solvents and high tensile strength	Foods (indigestible fiber), biomedical (wound healing, tissue engineered blood vessels) and audio speaker diaphragms	*Acetobacter* spp.
Succinoglycan	Glucose and galactose	5 × 10^3^–1 × 10^6^	High viscosity and acid stability	Food and oil recovery	*Alcaligenes faecalis* var. myxogenes
Glucuronan	Glucuronic acid	6 × 10^4^–6 × 10^5^	Gelling and thickening capacity	Food and cosmetics products	*Sinorhizobium meliloti* M5N1CS and *Gluconacetobacter hansenii*
Colanic acid	Fucose, glucose, glucoronate, and galactose	2 × 10^4^–6 × 10^5^	Gelling capacity	Cosmetics and personal care products	*E. coli*, *Shigella* spp., *Salmonella* spp. and *Enterobacter* spp.
